# “The Immune Conundrum”: Acquired Hemophilia A, Immune Thrombocytopenia, and Neutropenia in a Patient with Pancreatic Cancer

**DOI:** 10.1089/crpc.2015.29011.prg

**Published:** 2016-01-01

**Authors:** Praveen Ramakrishnan Geethakumari, Ashwin Sama, Jaime G. Caro, Charles J. Yeo, Srikanth Nagalla

**Affiliations:** ^1^ Department of Hematology and Medical Oncology, Thomas Jefferson University Hospital, Philadelphia, Pennsylvania.; ^2^ Department of Medical Oncology, Thomas Jefferson University Hospital, Philadelphia, Pennsylvania.; ^3^ Department of Hematology, Thomas Jefferson University Hospital, Philadelphia, Pennsylvania.; ^4^ Department of Surgery, Thomas Jefferson University Hospital, Philadelphia, Pennsylvania.

**Keywords:** acquired hemophilia A, immune thrombocytopenia, neutropenia, pancreatic cancer, paraneoplastic syndromes

## Abstract

**Background:** Malignancy-associated bleeding can pose diagnostic dilemmas. We report a unique case of paraneoplastic acquired hemophilia A (AHA), immune thrombocytopenia (ITP), and immune neutropenia in a patient with pancreatic adenocarcinoma.

**Case Presentation:** A 66-year-old male with newly diagnosed pancreatic cancer and normal preoperative hematological evaluation was taken to the operating room for pancreaticoduodenectomy. The operation was aborted due to empyema of the gall bladder, cholangitis, and local extent of disease. Postoperatively, the patient developed bleeding diatheses with mucocutaneous and intra-abdominal bleeding and a prolonged activated partial thromboplastin time. Evaluation revealed high-titer factor VIII inhibitor confirming AHA. Management with bypassing agents such as recombinant activated factor VII, factor VIII inhibitor bypassing activity, and immunosuppression with steroids, cyclophosphamide, and rituximab achieved remission in 2 months. ITP developed after achieving normal factor VIII levels, which was managed with intravenous immunoglobulin. Neutropenia was detected before initiation of chemotherapy and was managed with granulocyte-colony stimulating factor.

**Conclusion:** These unique challenges posed by paraneoplastic hematological syndromes warrant the need for astute clinical judgment and multidisciplinary collaboration for effective management.

## Introduction

Malignancy-associated bleeding can be multifactorial posing diagnostic dilemmas.^1^ Acquired hemophilia A (AHA) is a rare life-threatening disorder caused by autoantibodies to factor VIII that may occur spontaneously (50% of cases) or secondary to malignancy, pregnancy, autoimmune disease, or medications.^2,3^ Autoimmune thrombocytopenia and neutropenia are uncommon paraneoplastic syndromes.^4^

We describe a challenging presentation of acquired immune dysregulation causing three distinct autoimmune syndromes of AHA, immune thrombocytopenia (ITP), and immune neutropenia.

## Case Report

A 66-year-old Caucasian male presented with obstructive jaundice in January 2015. He was found to have a hypodense mass in the head of pancreas and underwent biliary stent placement. Endoscopic ultrasound and fine needle aspiration revealed adenocarcinoma of the pancreas. The tumor was staged as IB, cT2cN0cM0. The patient was scheduled for a Whipple procedure at the end of February. At surgical exploration, empyema of the gall bladder, pyobilia, and locally advanced tumor were discovered. Surgical resection was aborted and Roux-en-Y hepaticojejunostomy was performed for optimal biliary drainage. No excessive bleeding was encountered during surgery. Broad spectrum antibiotics were started intraoperatively and continued perioperatively.

**FIG. 1.**
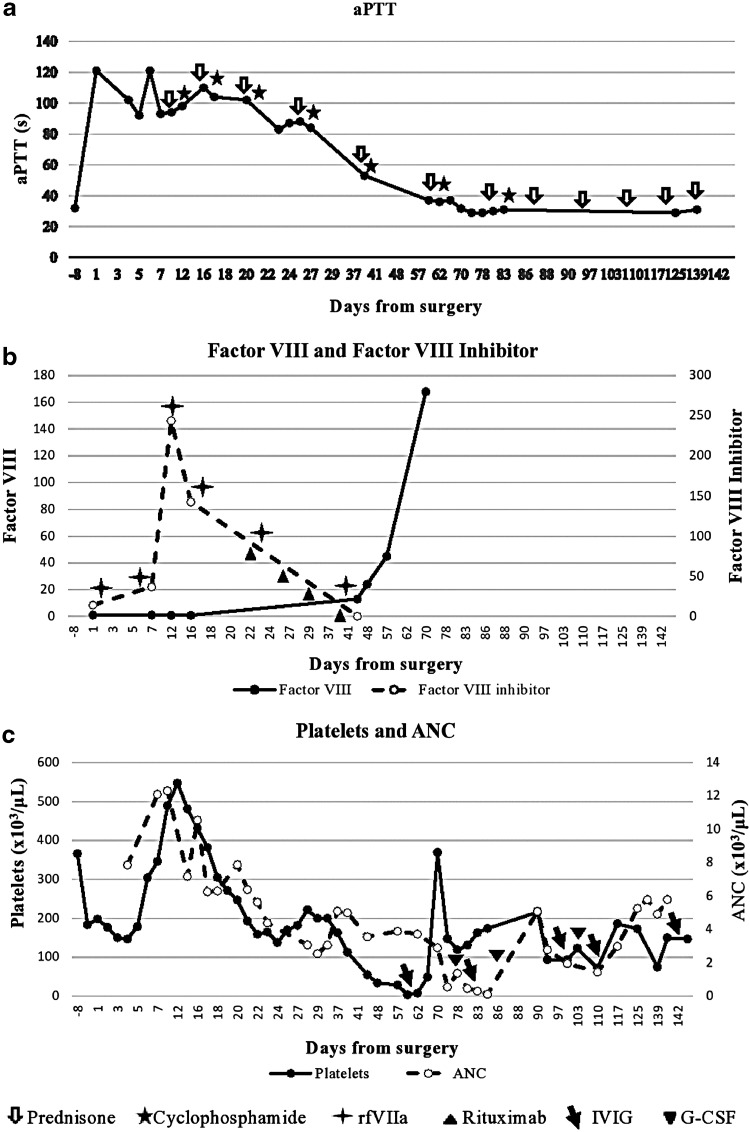
Trends of **(a)** activated partial thromboplastin time (aPTT), **(b)** factor VIII level and inhibitor titer, **(c)** platelet and absolute neutrophil count (ANC) and therapeutic interventions.

Preoperative hematological evaluation was within normal limits. Postoperatively, the patient developed bleeding from drains and intravenous line sites. Prothrombin time was mildly increased at 13.9 sec and activated partial thromboplastin time (aPTT) was elevated at 85 sec. A 1:1 aPTT mixing study did not correct, suggesting the presence of an inhibitor. Further analysis showed a factor VIII level of 1% and factor VIII inhibitor titer of 14.3 Bethesda units (BU). Specific tests such as lupus anticoagulant, dilute Russel viper venom test, thrombin time, and other coagulation factors were within normal limits (Fig. 1 and Table 1).

**Table 1.** **Normal Laboratory Values Compared to Immediate Post-Operative Values of Patient**

Normal laboratory values (general population)	
Hemoglobin, g/dL	14–17
White blood cell count, ×10^9^/L	4–11
Absolute neutrophil, ×10^9^/L	1.6–8
Platelet count, ×10^9^/L	140–400
Prothrombin time, sec	8.9–13.3
aPTT, sec	26–38
Factor VIII activity, %	41–159
Immediate postoperative laboratory values of patient *(Post-op Day 0)*	
aPTT, sec, *N* = 26–38	121
aPTT mix, sec	52.4
Factor VIII activity, %, *N* = 41–159	1
Factor IX activity, %, *N* = 50–160	97
Factor XI activity, %, *N* = 58–143	74
Factor XII activity, %, *N* = 48–158	59
Thrombin time, sec, *N* = 11.2–17.3	12.4
dRVVT, sec, *N* = 24.4–37.2	35.1
Factor VIII inhibitor, BU, *N* = <0.6 BU/mL	14.3

aPTT, activated partial thromboplastin time; BU, Bethesda units; dRVVT, dilute Russel viper venom test.

The patient developed abdominal wall ecchymoses, and multiple intra-abdominal hematomas were detected on axial CT imaging. His hemoglobin dropped from 13.7 to 7.4 g/dL and was supported with packed red blood cell (PRBC) transfusions. He was immediately started on recombinant activated factor VII (rFVIIa) and intravenous immunoglobulin (IVIG) therapies, which controlled bleeding but inhibitor titer increased to 37 BU. Cyclophosphamide was initiated at a dose of 2 mg/kg body weight (200 mg) daily. The inhibitor titer peaked at 243.9 BU and factor VIII at nadir was <1% (Fig. 1). He required 6 units of PRBCs. The patient was discharged on a regimen of prednisone and cyclophosphamide.

He was readmitted for gastrointestinal bleeding and symptomatic anemia. Factor VIII was still <1% and the inhibitor titer was 142.9 BU. A descending colonic source of presumed diverticular bleeding was identified and managed with PRBC transfusions and restarting rfVIIa. No interventional procedure was performed due to the risk of catastrophic bleeding. Rituximab was initiated at 375 mg/m^2^ weekly for four doses. Factor VIII inhibitor bypassing activity (FEIBA) was then started due to the frequent need of transfusions. He required 13 units of PRBCs. He was discharged on a regimen of rfVIIa, cyclophosphamide, prednisone, and completed four doses of rituximab. The factor VIII levels improved to 13% and inhibitor titer decremented to <0.6 BU (Fig. 1).

Moderate thrombocytopenia was detected on follow-up with platelet count of 35,000/μL. The cyclophosphamide dose was decreased to 150 mg daily. The platelet count dropped further to 4000/μL and the patient developed wet purpura in the oral cavity. As RBC and WBC counts were stable, he was initiated on IVIG for ITP, resulting in normalization of platelet count (Fig. 1). He was discharged on a regimen of cyclophosphamide and slow steroid taper.

Repeat laboratories showed a decline in WBC count to 200/μL, with a normal platelet count and hemoglobin. This was diagnosed as autoimmune neutropenia. The steroid dose was increased to 60 mg daily, cyclophosphamide stopped, and granulocyte-colony stimulating factor (G-CSF) initiated (daily for four doses), with prompt recovery of WBC counts (Fig. 1).

He was evaluated by a multidisciplinary team of hematology, and medical, radiation, and surgical oncology. After blood count recovery, a short course of radiation therapy was started for pancreatic cancer followed by chemotherapy.

## Discussion and Literature Review

Paraneoplastic hematological syndromes include AHA, ITP, autoimmune hemolytic anemia, antiphospholipid antibodies, granulocytosis, eosinophilia, thrombocytosis, anemia, and pure red cell aplasia.^4^ We are reporting the first patient, to the best of our knowledge, to develop AHA, ITP, and autoimmune neutropenia in a postoperative setting for pancreatic cancer. The other unique feature is the development of autoimmune cytopenias after the patient achieved normal factor VIII levels on multiple immunosuppressants.

The postulated mechanism of AHA is immune dysfunction from an abnormal T-cell response to a stimulatory antigen or abnormal interactions between T and B cells leading to autoantibody generation. Research has shown variations in the CTLA-4 gene and upregulation of B-cell activating factor in cases of AHA. Acquired factor VIII inhibitors exhibit type 2 kinetics with rapid initial inactivation of factor VIII followed by a slower phase of equilibrium, compared to alloantibodies in congenital hemophilia A that follow type I kinetics.^2,5^

AHA accounts for high-mortality rates ranging between 7% and 22%.^6^ Severe bleeding (spontaneous [75%] and provoked [25%]) and unexplained prolonged aPTT are the most common presentations. Spontaneous subcutaneous, muscular, and retroperitoneal bleeds are common, but mucosal and intracranial bleeds have been reported. Hemarthrosis is uncommon unlike congenital hemophilia. About 5–7% patients can present with isolated laboratory abnormalities.^2,3^

AHA can be associated with underlying malignancy in 6.7–14% patients. The prevalence of paraneoplastic factor VIII inhibitors was reported to be 11.8% from the European Acquired Hemophilia Registry (EACH2), 67.8% with solid tumors, and 32.2% with hematological malignancies. Among solid tumors, AHA has been reported more often in prostate, lung, and gastrointestinal cancers.^2,7,8^

About 20% of patients with paraneoplastic factor VIII antibodies develop these in conjunction with surgery for cancer. The majority of patients had gastrointestinal cancers and median age was 69 years.^9–13^ They were predominantly male, in contrast to female predominance in nonsurgical paraneoplastic factor VIII inhibitors from the EACH2 registry. Triggering of paraneoplastic AHA by surgery is unique and has only been observed in syndromes like autoimmune hemolytic anemia. The median duration from surgery to antibody detection was 3 months (range 1 week to 6 months). Most patients had low antibody titers (median 14 BU; range 1.7–64 BU).^14^ Our case is unique in the rapid occurrence of high-titer autoantibody to factor VIII following surgery. It is also possible that underlying pancreatic cancer,^15^ infection (cholangitis), or medications such as antibiotics and anesthetics during surgery triggered the abnormal antibody production.

Management of AHA is two pronged: (1) achievement of hemostasis and (2) eradication of the autoantibody production.^16^ Bypassing agents in the form of rFVIIa and FEIBA are the main treatment options for patients with major bleeding and high-titer inhibitors. Either agent has greater than 90% success rate when used as first-line therapy in AHA. If one agent fails to achieve adequate hemostasis, the other should be tried as in our case.

Immunosuppression to achieve inhibitor eradication can be complicated by the underlying infection as in our case. Current therapy involves the combination of steroids and cyclophosphamide that have success rates of up to 80%. Rituximab is increasingly used as first- and second-line strategies and showed a 59% complete remission (CR) rate as single agent in the EACH2 registry. Other strategies include azathioprine, mycophenolate mofetil, and calcineurin inhibitors.^17,18^

Our patient needed bypassing agents, IVIG, steroids, cyclophosphamide, and rituximab to achieve hemostasis and inhibitor eradication. Sequential and combined use of these agents must be tailored to the patient's presentation. Disease flares on immunosuppression withdrawal may necessitate reintroduction of these agents.

The major cause of cancer-associated thrombocytopenia is secondary to bone marrow aplasia from chemotherapy or radiation. Less common causes are marrow infiltration by tumor, disseminated intravascular coagulation, and related to drug. Paraneoplastic ITP has been associated with hematological malignancies and solid tumors. In a review of 68 cases of paraneoplastic ITP with solid tumors, it was commonly described in tumors of the lung, breast, kidney, and ovary, and rarely in prostate cancer. It has only been described in one case of pancreatic cancer before this report.^19^ ITP can occur concurrently with the cancer diagnosis (50%), preceding diagnosis (25%), and the rest manifest during disease course or at recurrence. Most patients responded to steroid treatment or splenectomy and only few had a CR after definitive surgical resection or chemotherapy for the cancer [4]. Our patient had already received steroids, rituximab, and was on cyclophosphamide in remission from AHA, before ITP developed and had good response to IVIG. It is interesting to observe the emergence of ITP on immunosuppression.

The neutropenia before chemotherapy initiation may be attributed to the cytotoxic effect of cyclophosphamide, but with normal platelet count, improving hemoglobin, and appropriate reticulocyte count, paraneoplastic autoimmune neutropenia seems the more likely diagnosis. The patient responded to G-CSF, and reinitiation of cyclophosphamide did not affect the WBC count. The treatment sequence for pancreatic cancer was modified in our case to start with radiation therapy, followed by chemotherapy once platelet and neutrophil counts stabilized.

## Conclusions

This case describes a unique *paraneoplastic immune conundrum* manifesting as three entities of AHA, ITP, and neutropenia in pancreatic cancer. This reiterates the need for astute clinical judgment and multidisciplinary collaboration for making accurate diagnoses and instituting prompt therapy for patients. Research in cancer immunology should better elucidate paraneoplastic phenomena improving future treatment options.

## Abbreviations Used

AHA: acquired hemophilia A
aPTT: activated partial thromboplastin time
BU: Bethesda units
CR: complete remission
dRVVT: dilute Russel viper venom test
EACH2: European Acquired Hemophilia Registry
FEIBA: factor VIII inhibitor bypassing activity
G-CSF: granulocyte-colony stimulating factor
ITP: immune thrombocytopenia
IVIG: intravenous immunoglobulin
PRBC: packed red blood cell
rFVIIa: recombinant activated factor VII
